# Superior mesenteric artery syndrome in severe anorexia nervosa: A case series

**DOI:** 10.1002/ccr3.2577

**Published:** 2019-12-11

**Authors:** Ashlie Watters, Dennis Gibson, Elizabeth Dee, Margherita Mascolo, Philip S. Mehler

**Affiliations:** ^1^ ACUTE Center for Eating Disorders at Denver Health and Hospital Authority Denver Colorado; ^2^ Department of Medicine University of Colorado School of Medicine Aurora Colorado; ^3^ Department of Radiology Denver Health and Hospital Authority Denver Colorado; ^4^ Alsana Thousand Oaks California

**Keywords:** anorexia nervosa, CT scan, gastroparesis, GI series, superior mesenteric artery syndrome

## Abstract

Superior mesenteric artery syndrome presents with nonspecific GI complaints, hindering weight restoration in those with anorexia nervosa. Diagnosis is made with radiologic testing, and treatment requires only weight restoration, negating the need for surgical intervention.

## Graphical abstract

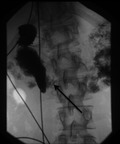

## 1 INTRODUCTION

Superior mesenteric artery (SMA) syndrome is a rare complication of severe malnutrition that leads to intestinal obstruction, causing postprandial epigastric abdominal pain and nausea.^1^ This syndrome was first described by Rokitansky in the mid‐19th century. It is defined as the extrinsic compression of the third part of the duodenum by the aorta posteriorly and the superior mesenteric artery anteriorly.^2^ The mesentery around the SMA is surrounded by a fatty tissue pad which forms a cushion that allows the duodenum to pass unobstructed between the SMA and the aorta. Weight loss and its resulting malnutrition lead to a loss of said fat pad surrounding the SMA, which allows for migration of the SMA medially, thus resulting in a narrowing of the normal angle between the SMA and the aorta. This narrowing results in external mechanical compression of the duodenum as it passes between the SMA and the aorta. The ensuing mechanical compression leads to symptoms similar to an intestinal obstruction, specifically abdominal pain and nausea that are aggravated by oral intake and relieved by emesis. The overall prevalence of SMA syndrome is unknown; however, it is estimated to be 0.013%‐3% in the general population based on barium studies.^3^ SMA syndrome can be the result of a multitude of medical conditions that lead to weight loss and a malnourished state, namely wasting diseases such as cancer, burns, AIDS, and chronic infections; malabsorptive states; or voluntary or involuntary lack of caloric intake such as patients with anorexia nervosa (AN).^4^

**Figure 1:**
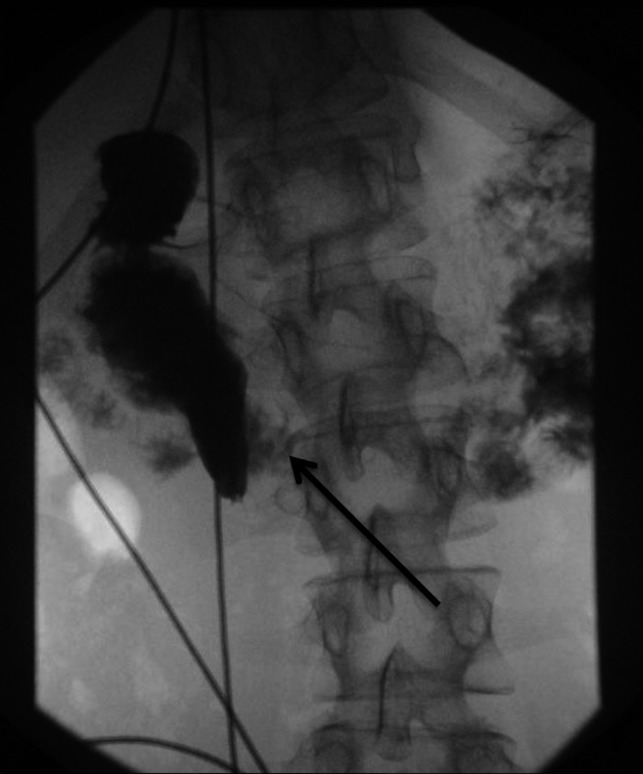
Image of an upper gastrointestinal (GI) single contrast study showing partial obstruction of the duodenum by the overlying superior mesenteric artery (SMA)

**Figure 2:**
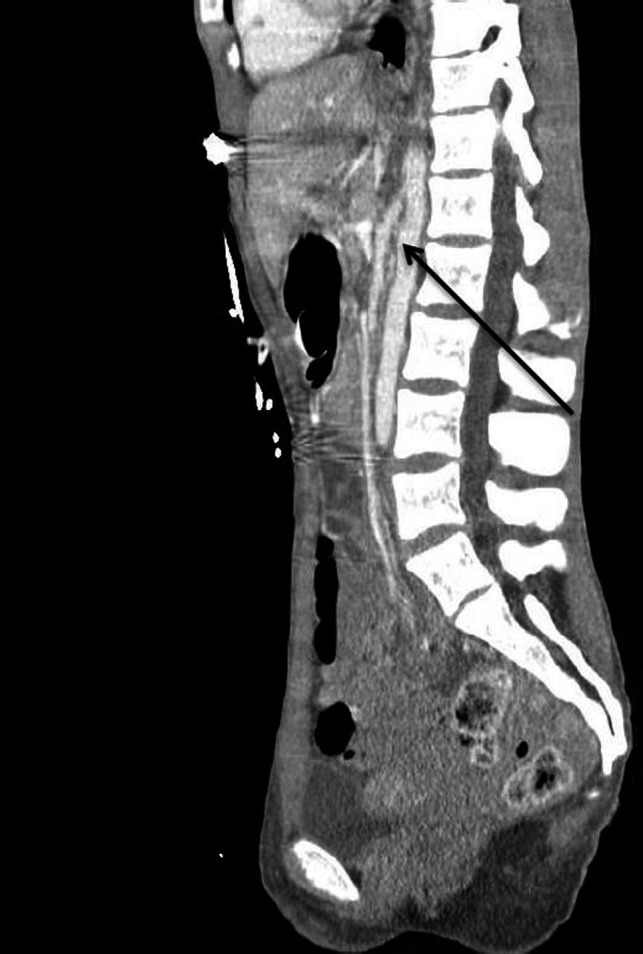
A sagittal image of a computed topography (CT) of the abdomen and pelvis with intravenous contrast showing the narrowed angle between the superior mesenteric artery (SMA) and the aorta

The diagnosis of SMA syndrome initially requires a high index of clinical suspicion based on the aforementioned symptoms. There are two radiologic imaging modalities that can be helpful in confirming the diagnosis.^5^ The first, an upper gastrointestinal (GI) series uses ingested barium while observing it as it transitions through the gastrointestinal tract (Figure 1). If there is complete SMA syndrome, the third part of the duodenum is obstructed by the new anatomic position of the SMA and no barium contrast material passes through when the patient is supine. In cases of partial SMA syndrome, even when the patient is supine, there is some minimal contrast material that passes through the third part of the duodenum. The second imaging modality used to confirm the diagnosis is a computerized tomography (CT) scan of the abdomen with intravenous contrast. The CT scan is able to directly visualize the anatomy of the aorta, SMA, and the duodenum and thus allows the radiologist to calculate the degrees of reduced aortic–mesenteric angle to confirm a mechanical obstruction (Figure 2).^6^ An angle <25° is highly suggestive, especially when the aortomesenteric distance is ≤8 mm. Abdominal X‐rays are often obtained in patients with AN given their vague abdominal complaints. They are not helpful per se in the diagnosis of SMA syndrome; however, they can show complications of this syndrome, such as acute gastric dilatation.^7^

The purpose of this case series is to highlight symptoms of SMA syndrome in patients with AN in order to avoid potential serious complications and to effectively avert impediments to the critically important process of rehabilitation of weight restoration. Furthermore, this case series highlights success in the treatment of SMA syndrome in AN with only the use of weight restoration and without need for surgical intervention.

## 2 METHODS

### 2.1 Study design

This is a retrospective case series designed to identify the clinical characteristics, symptoms, method of diagnoses, and treatment of SMA syndrome in patients with severe malnutrition secondary to AN. We reviewed records of patients admitted to the ACUTE Center for Eating Disorders (ACUTE) at Denver Health for medical stabilization of their severe AN between January 1, 2012, and December 31, 2014. ACUTE is a thirty bed, Center for Excellence, medical stabilization unit for the most medically compromised eating‐disordered patients as previously described.^8^ Patients were assessed and diagnosed with AN, restricting subtype (AN‐R) or binge–purge subtype (AN‐BP) according to criteria from the DSM IV and DSM‐V, by a licensed clinical psychologist upon admission. Patients diagnosed with AN‐R are underweight, fearful of weight gain and of food intake, and purely restrict calories. Those diagnosed with AN‐BP share the above characteristics and also engage in purging behaviors such as vomiting, laxative abuse, diuretic abuse, or a combination of these.^9^, ^10^ From there, patients were identified as having complete or partial SMA syndrome if the radiology report cited the diagnosis of SMA syndrome. Patients were excluded if they did not have an objective radiologic diagnosis of SMA syndrome. The study was approved by the Colorado Multiple Institutional Review Board.

During the study period, 292 AN‐R and AN‐BP patient records were reviewed. Of those patients, 16 were suspected to have SMA syndrome and eight of those patients were confirmed as having a diagnosis of SMA syndrome via radiological imaging while on the ACUTE unit.

### 2.2 Data

Manual chart review was conducted for clinical variables of interest including patient characteristics, mode of diagnosis (upper GI series or CT scan), complete or partial SMA syndrome diagnosis, presence of gastroparesis, symptoms consistent with SMA syndrome, treatment modalities, and resolution of symptoms. Anthropometrics were calculated upon admission and discharge. Percent ideal body weight (%IBW) was calculated using the Hamwi method,^11^ and body mass index (BMI) was calculated as weight in kilograms over height in meters squared.

## 3 RESULTS

All patients were female, average %IBW was 63.1% (SD = 10.1), and the average age of the group was 33 years (SD = 13.3), ranging from 19 to 51 years. Five patients were diagnosed with AN‐R, three with AN‐BP, and approximately one‐third admitted at <60% of their IBW. Table 1 lists the symptoms, mode of imaging, treatment, and basic demographics for each of the eight patients. In all cases, the impetus for workup of SMA syndrome was prompted by a high index of clinical suspicion based on the patient's symptoms. All patients complained of abdominal pain, six reported nausea, and two patients experienced emesis. Patients with relevant abdominal symptoms were further evaluated with either an upper GI series or a CT scan of the abdomen with intravenous contrast. The radiologic study modality pursued was mainly based on ease of timely availability by radiology to perform the study. Of the eight patients, six (75%) were confirmed to have SMA syndrome based on a CT scan and three (38%) were confirmed through an upper GI series (one patient received both modalities). Concurrent gastroparesis was diagnosed clinically in all eight patients. Given the severity of their symptoms and findings from the radiologic examination, all eight patients were treated by halting the ingestion of regular food and, instead, were weight restored with a progressive oral liquid and soft diet. Moreover, in view of the severity of their symptoms, three of the eight patients required supplemental calories via the insertion of a gastric or nasojejunal feeding tube in addition to their oral liquid diet. Six (75%) of the patients had complete resolution of symptoms prior to discharge from ACUTE and were ingesting a regular diet, and the remaining two (25%) patients had marked improvement of symptoms characterized by reduction in nausea and pain following oral caloric intake, as these patients were still ingesting a soft diet. All patients, aside from one, discharged to a residential eating disorder treatment facility. The other patient discharged home against the treatment team's medical advice.

**Table 1:** Patient demographics, treatment, and clinical outcomes

ID	Age (yrs)	Subtype of AN	Admission %IBW	Admission BMI (kg/m^2^)	Symptoms of SMA	Mode of imaging	Treatment†	LOS (days)	Discharge %IBW	Discharge BMI (kg/m^2^)	Discharge symptoms
A	40	AN‐R	64.8	13.2	Postprandial bloating, nausea, gas, and postprandial epigastric pain	CT, GI	PEJ tube placed by surgery, plus oral and liquid kcals; oral and liquid diet advanced as tolerated	41	72.3	14.7	Improvement in pain
B	24	AN‐R	50.6	10.5	Nausea/severe abdominal pain postmeals; liquids preferred	GI	NJ tube placed and then GJ tube by IR; oral liquid diet	28	66.1	13.8	Improvement in pain
C	47	AN‐R	51.3	10.7	Postprandial abdominal pain/bloating/distention	CT	Liquid diet	27	60.1	12.5	Resolved
D	19	AN‐BP	54.3	10.9	Bloating/early fullness/severe abdominal pain; relieved by emesis	CT	Liquid diet	28	71.4	14.4	Resolved
E	22	AN‐BP	67.4	14.0	Early satiety; postprandial abdominal pain and nausea relieved by emesis	CT	PEG tube placed by surgery, then converted to PEJ tube, then back to PEG and some oral liquids	35	74.7	15.5	Resolved
F	19	AN‐R	65.2	13.6	Extreme sharp abdominal pain and nausea postprandial	GI	Liquid diet	21	70.0	14.6	Resolved
G	51	AN‐BP	75.0	15.1	Abdominal pain	CT	Liquid diet	12	81.2	16.3	Resolved
H	42	AN‐R	76.5	15.6	Abdominal pain and constant nausea	CT	Liquid diet then transitioned to different consistencies of solid foods	21	84.5	17.2	Resolved

Abbreviations: %IBW, Percent ideal body weight; AN‐BP, Anorexia nervosa binge–purge subtype; AN‐R, Anorexia nervosa restricting subtype; BMI, Body mass index; CT, Computer tomography; GI, Gastrointestinal series; GJ, Gastrojejunostomy; IR, Interventional radiology; LOS, Length of Stay; NJ, Nasojejunal; PEG, Percutaneous endoscopic gastrostomy; PEJ, Percutaneous endoscopic Jejunostomy; SMA, Superior mesenteric artery.

^†^ The NJ tube in Patient B was converted to a GJ tube due to ongoing nausea; therefore, bypassing the duodenal obstruction and the PEJ tube for Patient E was ultimately converted back to a PEG tube due to inability of the feeding tube to stay in place after multiple attempts.

## 4 DISCUSSION

Superior mesenteric artery syndrome is characterized by postprandial abdominal pain and nausea that begins 15‐20 minutes after ingestion of oral food and is relieved by emesis or the passage of a few hours. In our case series, liquids or soft foods were better tolerated, especially when the obstruction is incomplete. The symptoms that patients experience lead to further decreases in oral intake, which in turn brings about further weight and fat pad loss and thus worsening of the duodenal obstruction. In the eating disorder population, the symptoms of SMA syndrome can be inappropriately overlooked or dismissed and instead attributed to the inherent psychiatric component of this disease and the aversion to food and weight gain.^12^ Also, patients with AN often have co‐morbid conditions such as gastroparesis that can aggravate abdominal symptoms and make ongoing attempted oral intake very challenging. Gastroparesis, or slowed gastric emptying, manifests in patients with more severe forms of AN with symptoms of bloating, nausea, and early satiety, but not actual pain.^13^, ^14^

In patients diagnosed with AN, SMA syndrome presented with symptoms of postprandial abdominal pain and for some, nausea relieved by emesis. On review of documented symptoms and complaints, patients also endorsed fullness and early satiety as well as bloating following any oral intake. When asked about consistency of foods, most patients stated that solids caused worse symptoms and for a much longer period of time after eating than did liquids.

There is no pathognomonic physical examination finding or sign of SMA syndrome. The physical examination in this patient population was reflective of their severely malnourished state. Given the average %IBW of 63.1%, patients were cachectic but overall had nonsurgical abdominal examinations. Thus, there was no rebound or guarding present, although there was occasional mild abdominal tenderness on palpation.

Treatment of SMA syndrome necessitates gaining enough weight to ultimately reconstitute the fat pad between the aorta and the SMA and re‐tether the SMA in its normal, lateral position in order to alleviate the mechanical obstruction on the duodenum. Thus, nutritional rehabilitation becomes the cornerstone of treatment with close monitoring and correction of electrolytes during the early stages of nutritional support to decrease the likelihood of development of refeeding syndrome. Patients with partial SMA syndrome, or duodenal narrowing with incomplete compression, can be treated with liquid oral calories with advancement to soft foods and eventually full solids as symptoms improve. This can be accomplished through placement of a feeding tube, potentially bypassing the area of narrowing, pending the severity of symptoms. However, complete SMA syndrome, or total compression of the duodenum, will often require parental nutrition until the narrowing alleviates enough that enteral nutrition can be tolerated. There can be an increased risk of surgical complication when malnutrition is present; therefore, surgical intervention should not be a consideration unless weight restoration fails to resolve the symptoms.

All eight patients had partial or complete obstruction of the SMA as evidenced by radiologic examination. As such, these patients were treated in the most conservative approach which consisted of a liquid diet as long as symptomatically they were able to tolerate treatment. All patients were treated with oral liquid calories, and three had the addition of a feeding tube in order to meet the caloric demands that allowed weight gain. There is no consensus regarding the minimal weight gain that alleviates symptoms of SMA syndrome. A progressive oral diet should be continued, and calories should be increased in an effort to continue to gain weight until symptoms resolve. In our cohort, six patients had complete resolution of their symptoms prior to discharge, while the remaining two had partial alleviation of symptoms, which included improvement in pain. Motility agents such as metoclopramide and macrolide antibiotics can also be administered to patients with partial SMA syndrome to facilitate gastric emptying^15^, ^16^ and improve the symptoms of gastroparesis, thus improving tolerance of the oral liquid calories and resolution of the SMA syndrome^17^; however, it is the recommendation of these authors to avoid these agents in individuals with complete SMA syndrome.

In summary, this case series highlights the fact that the diagnosis of SMA syndrome requires a high index of suspicion in the correct clinical scenario, in which severely malnourished patients with AN present with postprandial nausea and abdominal pain alleviated by emesis. In the eating disorder population, these symptoms should not be dismissed and solely attributed to the underlying psychiatric disorder. SMA syndrome can be accurately diagnosed and confirmed by radiological modalities such as an upper GI series or CT scan. As long as the duodenal obstruction is incomplete, patients can safely be treated with a progressive oral liquid diet until symptoms resolve or are alleviated, at which point diet consistency can be advanced as tolerated.

## CONFLICT OF INTEREST

The authors declare no conflict of interest.

## AUTHOR CONTRIBUTIONS

AW: conducted chart review and drafted the manuscript. DG: drafted and revised the manuscript. ED: performed clinical diagnosis. MM: drafted and revised the manuscript. PM: drafted, revised, and reviewed the manuscript.
